# Seasonal Variation of the Chemical Composition and Antimicrobial and Cytotoxic Activities of the Essential Oils from *Inga laurina* (Sw.) Willd.

**DOI:** 10.3390/molecules19044560

**Published:** 2014-04-11

**Authors:** Fabiana B. Furtado, Francisco J. T. de Aquino, Evandro A. Nascimento, Carla de M. Martins, Sérgio A. L. de Morais, Roberto Chang, Luís C. S. Cunha, Luís F. Leandro, Carlos H. G. Martins, Mário M. Martins, Claudio V. da Silva, Fabrício C. Machado, Alberto de Oliveira

**Affiliations:** 1 Laboratory of Natural Products and Chromatography, Chemistry Institute, Federal University of Uberlândia, Uberlândia, Minas Gerais 38408-144, Brazil; E-Mails: fabisbarcelos@hotmail.com (F.B.F.); aquino@iqufu.ufu.br (F.J.T.A.); eanascimento@ufu.br (E.A.N.); salemos@ufu.br (S.A.L.M.); chang@iqufu.ufu.br (R.C.); luisscunha@gmail.com (L.C.S.C.); mariomm1988@yahoo.com.br (M.M.M.); 2 Federal Institute Goiano, Morrinhos, Goiás 75650-000, Brazil; E-Mail: carla.martins@ifgoiano.edu.br; 3 Nucleus of Research in Sciences and Technology, Laboratory of Research in Applied Microbiology (LaPeMA), University of Franca, Franca, São Paulo 14404-600, Brazil; E-Mails: luis.fernando.leandro@hotmail.com (L.F.L.); carlos.martins@unifran.edu.br (C.H.G.M.); 4 Institute of Biomedical Sciences, Laboratory of Trypanosomatids, Federal University of Uberlândia, Uberlândia, Minas Gerais 38400-902, Brazil; E-Mails: silva_cv@yahoo.com.br (C.V.S.); fabriciomachado@live.com (F.C.M.)

**Keywords:** *Inga laurina* (Sw.) Willd., Leguminosae, essential oil, antimicrobial activity, cytotoxic activity

## Abstract

The seasonal chemical composition of essential oils from *Inga laurina* was determined by GC/MS. In the stem bark’s essential oil extracted during the dry season, the presence of terpenoids (30.05%) stood out, and phytol (9.76%) was the major compound identified. For the stem bark oil obtained during the rainy season, in addition to terpenoids (26.63%), a large amount of fatty acids (46.84%) were identified, in particular palmitic acid (25.40%). Regarding the leaves’ essential oil obtained in the dry season, esters (42.35%) were the main components. The main ester present was (*Z*)-hex-3-enyl benzoate (10.15%) and the major compound of this oil was (*Z*)-hex-3-en-1-ol (14.23%). Terpenoids (33.84%), long-chain alkanes (27.04%) and fatty acids (21.72%) were the main components of the essential oil from leaves in the rainy season. Phytol (33.21%), nonacosane (21.95%) and palmitic acid (15.20%) were the major compounds identified. The antimicrobial activity against aerobic and anaerobic oral bacteria was evaluated by the microdilution broth method and cytotoxic activity was carried out with Vero cells. The essential oils from the rainy season showed a better inhibition of the bacterial growth with Minimal Inhibitory Concentrations (MIC) values of 25 or 50 µg·mL^−1^ for aerobic bacteria, and high selectivity against bacteria was observed. The large amount of fatty acids in rainy season oils may be related to the better inhibitory effects observed.

## 1. Introduction

*Inga laurina* (Sw.) Willd. belongs to the *Inga* genus (Leguminosae) and is popularly known as in Brazil as Angá or Ingá Branco. It is a tropical arboreal plant with a widespread distribution in Central and South America. Several species of *Inga*are used in folk medicine as an anti-inflammatory, an antidiarrheal and a nasal decongestant, and for skin treatment, earache and cleaning teeth [1,2]. Studies of these species have reported the isolation of some metabolites such as depsides [3], metabolites of nitrogen [4], pipecolic acids [5,6], steroidal glycosides [7] and phenolic compounds [1,7,8,9,10,11]. However, there are few chemical studies available related to *I. laurina* species [12]. *I. laurina* extracts have shown antioxidant [13] and antiplasmodial activities [14]. Additionally, a proteolytic inhibitor was found in their seeds and exhibited inhibitory activity of the trypsin enzyme [12,15], thus acting in this way as a pest control against *Homalinotus coriaceus*, *Diatraea saccharalis* and *Heliothis virescens* [16,17]. Species of the *Inga* genus are practically unexplored regarding the characterization of its essential oils and their biological activities. Amongst the *Inga* species studied, only the chemical composition of the essential oil from flowers of *Inga edulis* Mart. has been described in the literature [18].

The essential oils of many different plant species have shown interesting biological activities, such as antispasmodic, antinociceptive, antioxidant, anti-inflammatory, immunomodulatory, psychotropic, acaricide and expectorant effects [19]. Antidiabetic, antiviral and antitumoural activities have also been observed [20]. In addition to these effects, essential oils have shown significant antimicrobial properties against several Gram-positive and Gram-negative bacteria [20], including oral pathogens [21,22].

Thereby, the aim of the present study was to determine the chemical composition of the essential oils from leaves and bark of *I. laurina* in different seasons (dry and rainy) and to investigate the antimicrobial potential of the essential oils against aerobic and anaerobic oral pathogens and their cytotoxic effects against Vero cells.

## 2. Results and Discussion

### 2.1. Yield and Chemical Composition of the Essential Oils

For the essential oil of the bark, the yields were 0.34‰ ± 0.04‰ (*w*/*w*) in the dry and 0.49‰ ± 0.17‰ (*w*/*w*) in the rainy seasons. For the leaf oils, the yields were 3.71‰ ± 0.98‰ (*w*/*w*) in the dry and 3.07‰ ± 1.10‰ (*w*/*w*) in the rainy season. No significant differences were observed in the yields between essential oils from the bark or leaves with respect to the period of collection, but the yield of the essential oil from leaves was higher than the bark in the dry and rainy season. These results are similar to the reported for essential oil from *Caesalpinia echinata*, another Leguminosae species [23].

Table 1 shows the composition of the essential oil from bark and leaves of *I. laurina* in the dry season. The chemical class distribution of the volatile constituents of *I. laurina* in the dry season is summarized in Table 2.

**Table 1:** Chemical composition of the essential oil from the stem bark and leaves of *I. laurina* in the dry season.

Compound	AI Reference		AI Calculated		Composition % TIC			
					Stem bark		Leaves	
Ethyl butanoate	802 ^b^		807		3.03		-	
( *E*)-Hex-3-en-1-ol	852 ^a^		853		-		1.09	
( *Z*)-Hex-3-en-1-ol	853 ^a^		856		-		14.23	
Hexan-1-ol *	863 ^b^		868		-		5.69	
Benzyl alcohol *	1034 ^a^		1041		-		1.46	
Linalool *	1100 ^a^		1105		5.17		-	
Hotrienol	1104 ^a^		1108		-		1.74	
(3 *E*,6*Z*)-Nona-3,6-dien-1-ol	1160 ^a^		1163		-		1.43	
3-Hexenyl butanoate	1184 ^b^		1188		-		7.60	
Hexyl butanoate	1188 ^a^		1194		-		1.95	
*α*-Terpineol *	1195 ^a^		1199		4.18		-	
Methyl salicylate *	1196 ^a^		1200		-		4.16	
( *Z*)-Hex-3-enyl 2-methylbutanoate	1229 ^b^		1235		-		2.06	
Geraniol *	1254 ^a^		1263		3.71		-	
( *Z*)-Hex-3-enyl hexanoate	1378 ^b^		1383		-		8.29	
Hexyl hexanoate	1382 ^b^		1388		-		3.47	
( *E*)-Hex-2-enyl hexanoate	1391 ^a^		1391		-		0.91	
N.I.	-		1436		-		2.19	
N.I.	-		1443		3.94		-	
N.I.	-		1568		-		1.70	
( *Z*)-Hex-3-enyl benzoate	1572 ^a^		1576		-		10.15	
Hexyl benzoate	1579 ^b^		1582		-		2.03	
( *E*)-Hex-2-enyl benzoate	1587 ^b^		1590		-		1.73	
Hexadecane *	1600 ^b^		1600		4.57		-	
Oxygenated sesquiterpene	-		1608		3.75		-	
*γ*-Eudesmol	1630 ^b^		1640		3.48		-	
N.I.	-		1670		-		1.32	
N.I.		-		1671		4.33		-	
Heptadec-8-ene		1677 ^a^		1678		7.66		-	
Heptadecane *		1700 ^b^		1700		2.79		-	
N.I.		-		1708		4.88		-	
Pentadecanal		1717 ^a^		1715		-		3.69	
N.I.		-		1773		1.85		-	
2-Ethylhexyl salicylate		1807 ^b^		1810		3.25		-	
Hexahydrofarnesyl acetone (phytone)		1843 ^a^		1846		3.90		1.24	
Hexadecanol		1874 ^b^		1883		8.34		-	
Heptadecadienal		-		1889		-		1.78	
3,3,5-Trimethylcyclohexyl salicylate (Homosalate)		-		1891		4.60		-	
N.I.		-		1895		-		4.29	
Phytol *		2114 ^a^		2116		9.76		2.58	
N.I.		-		2154		3.43		-	
N.I.		-		2203		3.01		-	
N.I.		-		2265		3.41		-	
N.I.		-		2308		3.22		-	
Pentacosane *		2500 ^b^		2500		3.16		-	
Heptacosane *		2700 ^b^		2700		-		2.66	
Nonacosane *		2900 ^b^		2900		-		9.77	
				**Total (%):**		**99.42**		**99.21**	

N.I. = not identified; TIC = total ions chromatogram; AI = arithmetic index; * compound was injected and added to our library of standards. ^a^ NIST: Standard Reference Data [24]. ^b^ Adams mass spectral-retention index library [25].

**Table 2:** Chemical class distribution of the essential oil components from the stem bark and leaves of *I. laurina* in the dry season.

Functional groups	Stem bark (%)	Leaves (%)
Alcohols	8.34 (1)	23.90 (5)
Esters	10.88 (3)	42.35 (10)
Aldehydes	-	5.47 (2)
Ketones	3.90 (1)	1.24 (1)
Oxygenated monoterpenes	13.06 (3)	1.74 (1)
Oxygenated sesquiterpenes	7.23 (2)	-
Oxygenated diterpenes	9.76 (1)	2.58 (1)
Long-chain alkanes	10.52 (3)	12.43 (2)
Long-chain alkenes	7.66 (1)	-
N.I.	28.07 (8)	9.50 (4)

N.I. = not identified; numbers in parentheses refer to the compounds identified for each function.

**Figure 1:**
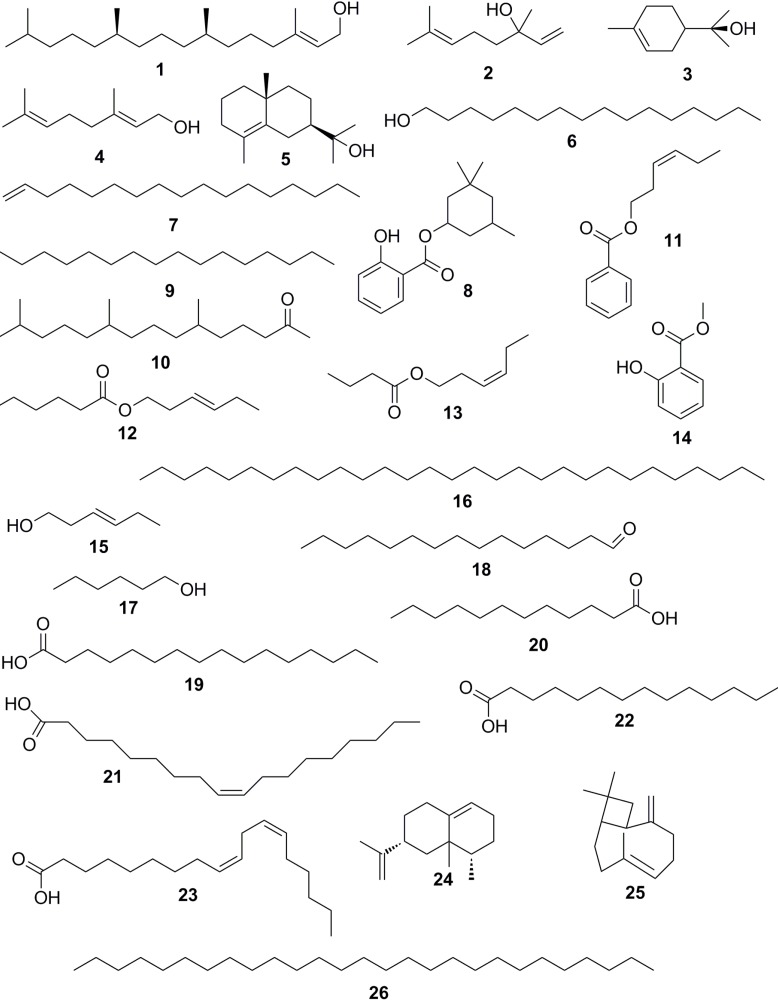
Structures of some compounds identified in the essential oils from *I. laurina.* **1**. Phytol; **2**. linalool; **3**. *α*-terpineol; **4**. geraniol; **5**. *γ*-eudesmol; **6**. hexadecanol; **7**. heptadec-8-ene; **8**. homosalate; **9**. hexadecane; **10**. phytone; **11**. (*Z*)-hex-3-enyl benzoate; **12**. (*Z*)-hex-3-enyl hexanoate; **13**. 3-hexenyl butanoate; **14**. methyl salicylate; **15**. (*Z*)-hex-3-en-1-ol; **16**. nonacosane; **17**. hexanol; **18**. pentadecanal; **19**. palmitic acid; **20**. lauric acid; **21**. oleic acid; **22**. myristic acid; **23**. linoleic acid; **24**. eremophylene; **25**. 9-epi-(*E*)-caryophyllene; **26**. heptacosane.

For the essential oil of the bark in the dry season, a total of 14 compounds were identified (Table 1). Terpenoids accounted for 30.05% of the compounds analysed (Table 2). Phytol (**1**, 9.76%) was the most abundant terpenoid. Other terpenoids found in relevant concentrations were linalool (**2**, 5.17%), *α*-terpineol (**3**, 4.18%), geraniol (**4**, 3.71%) and *γ*-eudesmol (**5**, 3.48%) (Figure 1). In addition to terpenoids, the major compounds identified in this essential oil were hexadecanol (**6**, 8.34%), heptadec-8-ene (**7**, 7.66%), homosalate (**8**, 4.60%), hexadecane (**9**, 4.57%) and phytone (**10**, 3.90%) (Figure 1).

In the leaves’ essential oil, a total of 22 compounds were identified for the same season (Table 1). Most of the compounds identified were esters, representing 42.35% of the total, while the bark’s essential oil accounted for 10.88% (Table 2). The plants usually utilize volatile esters in their chemical communication system and these compounds also act in defence mechanisms against pathogens [26]. The main esters present were the (*Z*)-hex-3-enyl benzoate (11, 10.15%), (*Z*)-hex-3-enyl hexanoate (12, 8.29%), 3-hexenyl butanoate (13, .60%) and methyl salicylate (14, 4.16%). In addition to the esters, the main compounds were the (*Z*)-hex-3-en-1-ol (15, 14.23%), nonacosane (16, 9.77%), hexanol (17, 5.69%) and pentadecanal (18, 3.69%) (Figure 1). Terpenoids accounted only for 4.32% (Table 2). Phytol (1) (2.58%) was the major terpenoid identified. Table 3 shows the composition of the essential oil from leaves and bark of *I. laurina* in the rainy season. The chemical class distribution of the volatile constituents of *I. laurina* in the rainy season is summarized in Table 4.

**Table 3:** Chemical composition of the essential oil from the stem bark and leaves of *I. laurina* in the rainy season.

Compound		AI Reference		AI Calculated		Composition % TIC		
						Stem bark		Leaves
4-Methyhexan-3-one		-		840		1.19		-
( *Z*)-Hex-3-en-1-ol		853 ^a^		856		-		9.59
Hexan-1-ol *		863 ^b^		868		-		0.70
N.I.		-		979		1.21		-
N.I.		-		1001		1.18		-
Linalool *		1100 ^a^		1105		2.69		-
Oxygenated monoterpenes		-		1140		7.80		-
3-Hexenyl butanoate		1184 ^b^		1188		-		0.59
( *Z*)-Hex-3-enyl hexanoate		1378 ^b^		1383		-		0.37
Eremophylene		1464 ^a^		1467		1.51		-
9-epi-( *E*)-Caryophyllene		1468 ^a^		1473		1.36		-
Sesquiterpene		-		1491		1.40		-
( *E*)-Nerolidol *		1564 ^a^		1568		-		0.28
Dodecanoic acid (lauric acid) *		1568 ^b^		1575		5.80		0.43
Tetradecanal		1611 ^b^		1611		0.94		-
Oxygenated sesquiterpene		-		1637		1.27		-
N.I.		-		1644		1.29		-
N.I.		-		1668		1.83		-
Heptadec-8-ene		1677 ^a^		1678		3.48		-
Heptadecane *		1700 ^b^		1700		1.68		-
Oxygenated sesquiterpene		-		1706		1.59		-
Oxygenated sesquiterpene		-		1713		2.01		-
N.I.		-		1714		-		0.43
Oxygenated sesquiterpene		-		1746		5.61		-
Tetradecanoic acid (myristic acid) *		1775 ^a^		1771		4.06		0.32
Hexahydrofarnesyl acetone (phytone)		1843 ^a^		1846		1.99		0.61
Hexadecanol		1874 ^b^		1883		2.15		0.37
3,3,5-Trimethylcyclohexyl salicylate (homosalate)		-		1891		3.87		-
N.I.	-		1919		-		0.99		
N.I.	-		1941		1.16		-		
Isophytol	1946 ^b^		1947		-		0.35		
Hexadec-9-enoic acid (palmitoleic acid)	1957 ^c^		1948		1.58		-		
N.I.	-		1962		1.04		-		
Hexadecanoic acid (palmitic acid) *	1970 ^a^		1972		25.40		15.20		
N.I.	-		2082		-		0.87		
N.I.	-		2099		-		0.80		
Phytol *	2114 ^a^		2116		1.39		33.21		
(9 *Z*,12*Z*)-Octadeca-9,12-dienoic acid (linoleic acid) *	2132 ^b^		2140		3.58		1.19		
( *Z*)-Octadec-9-enoic acid (oleic acid) *	2141 ^b^		2144		5.52		2.33		
N.I.	-		2149		-		1.28		
Octadecanoic acid (stearic acid) *	2170 ^a^		2169		0.90		2.25		
Tricosane *	2300 ^b^		2300		-		0.43		
Pentacosane *	2500 ^b^		2500		0.89		-		
Heptacosane *	2700 ^b^		2700		0.69		3.33		
Octacosane *	2800 ^b^		2800		-		1.33		
Nonacosane *	2900 ^b^		2900		-		21.95		
Triacontane *	3000 ^b^		3000		0.95		-		
			Total (%)		99.01		99.20		

N.I. = not identified; TIC = total ions chromatogram; AI = arithmetic index; * compound was injected and added to our library of standards. ^a^ NIST: Standard Reference Data [24]. ^b^ Adams mass spectral-retention index library [25]. ^c^ The Pherobase: Database of insect pheromones and semiochemicals [27].

**Table 4:** Chemical class distribution of the oil components from the stem bark and leaves of *I. laurina* in the rainy season.

Functional groups	Stem bark (%)	Leaves (%)
Alcohols	2.15 (1)	10.66 (3)
Esters	3.87 (1)	0.96 (2)
Aldehydes	0.94 (1)	-
Ketones	3.18 (2)	0.61 (1)
Oxygenated monoterpenes	10.49 (2)	-
Sesquiterpenes	4.27 (3)	-
Oxygenated sesquiterpenes	10.48 (4)	0.28 (1)
Oxygenated diterpenes	1.39 (1)	33.56 (2)
Long chain alkanes	4.21 (4)	27.04 (4)
Alkenes	3.48 (1)	-
Fatty acids	46.84 (7)	21.72 (6)
N.I.	7.71 (6)	4.37 (5)

N.I. = not identified; numbers in parentheses refer to the compounds identified for each function.

For bark’s essential oil from the rainy season, a total of 21 compounds were identified (Table 3). Most of them were fatty acids, representing 46.84% of the total oil (Table 4). These fatty acids were not observed in the bark’s oil obtained in the dry season and they did not result from the degradation of the esters present in it (Table 1). Palmitic (**19**, 25.40%), lauric (**20**, 5.80%), oleic (**21**, 5.52%), myristic (**22**, 4.06%) and linoleic (**23**, 3.58%) were the major fatty acids identified. The terpenoids represented 26.63% and were present in equivalent percentage (30.05%) when compared to the essential oil of the dry season (Table 2 and Table 4). The major terpenoids identified were linalool (**2**, 2.69%), eremophylene (**24**, 1.51%), phytol (**1**, 1.39%) and 9-epi-(*E*)-caryophyllene (**25**, 1.36%). Others representative compounds were homosalate (**8**, 3.87%) and heptadec-8-ene (**7**, 3.48%) (Figure 1).

With regards to the essential oil from leaves in the rainy season, 19 compounds were identified (Table 3). The terpenoids accounted for 33.84% and were found in higher amount when compared with oil obtained during the dry season (4.32%) (Table 2 and Table 4). The synthesis of some terpenoids occurs specifically in leaf structures called glandular trichomes [28] and studies have shown that in some plants the full development of these structures is light-dependent [29,30]. A greater or lesser light intensity can explain the variation of terpenoids (Table 2 and Table 4) when oils from leaves of *I. laurina* are compared at different collection periods. Already in bark, the synthesis of terpenoids is probably not as dependent on trichomes, and these compounds may be being produced and stored in another structures and probably for this, their concentrations were approximately constant in the different periods analyzed (Table 2 and Table 4). Phytol (**1**, 33.21%) was the major compound identified in this oil. The others major compounds identified in this essential oil were nonacosane (**16**, 21.95%), palmitic acid (**19**, 15.20%), (*Z*)-hex-3-en-1-ol (**15**, 9.59%) and heptacosane (**26**, 3.33%) (Figure 1). In addition, fatty acids (21.72%) and long-chain alkanes (27.04%) were the main class of components identified in this oil, comprising 82.60% of the total (Table 4). Esters content was below 1%, which is a big difference when compared to their content in the dry season (42.35%) (Table 2 and Table 4). Due to its chemical composition, hydrocarbons, alcohols and esters stand out as the main protective barrier against water loss by excessive sweating, action of pathogens, solar radiation and inputs of chemicals and contaminants [31]. The concentration of these classes of compounds was higher in the dry season (Table 2), which leads us to believe that these compounds play a protective role in this plant, especially in the leaves, a tissue more subject to loss of water and external injuries when compared to the bark.

In different seasonal periods, the plant synthesizes different compounds according to environmental conditions [32]. The data presented here demonstrate this fact (Table 1 and Table 3).

This is the first report showing the chemical composition of the essential oils of *I. laurina*. In the *Inga* genus, only the chemical composition of the essential oil from flowers of *I. edulis* Mart. has been previously described in the literature. In this study, the major components identified were linalool (**2**, 20%), tricosane (11.4%), palmitic acid (**19**, 7.6%) and other compounds in smaller proportions such as *α*-terpineol (**3**, 3.5%), geraniol (**4**, 3.14%), methyl salicylate (**14**, 2.7%), pentacosane (1.5%), benzyl alcohol (0.6%), heptacosane (**26**, 0.2%) and (*E*)-nerolidol (0.1%) [18]. All these compounds were also found in the essential oil of bark or leaf of *I. laurina* in a higher or smaller proportion. Plants have several secondary metabolic pathways that lead to the formation of compounds whose distribution is restricted to certain families, genera or species [33]. It is possible that species of this genus share metabolic pathways that lead to the synthesis of the same secondary metabolites due to the similarity observed when compared to the chemical constitution of the essential oils of *I. laurina* and *I. edulis*, although the analysed oils were extracted from different parts of these species.

### 2.2. Antimicrobial and Cytotoxic Activities of the Essential Oils

The antimicrobial activity of the essential oil from the bark and leaves in different seasons (dry and rainy) was determined against aerobic and anaerobic oral microorganisms. The results of antimicrobial activity and cytotoxicity are shown in Table 5.

**Table 5:** Inhibitory effect on the growth of aerobic and anaerobic oral bacteria (MIC values, μg·mL^−1^) and cytotoxic concentration (EC_50_, μg·mL^−1^) of the essential oil from the stem bark and leaves of *I. laurina* extracted in the dry and rainy seasons.

		Sample/Season				
		Dry		Rainy		
Microorganisms		Stem bark	Leaves	Stem bark	Leaves	CHD *
Anaerobic	*Porphyromonas gingivalis* ^a^ ATCC 33277	100	100	100	50	3688
	*Prevotella nigrescens* ^a^ ATCC 33563	200	100	400	100	1844
	*Fusobacterium nucleatum* ^a^ ATCC 25586	>400	>400	400	200	3688
	*Actinomyces naeslundii* ^b^ ATCC 19039	>400	>400	>400	400	1844
	*Bacteroides fragilis* ^a^ ATCC 25285	>400	>400	>400	>400	1475
Aerobic	*Streptococcus mutans* ^b^ ATCC 25175	200	200	25	50	0922
	*Streptococcus sanguinis* ^b^ ATCC 10556	200	100	50	50	3688
	*Streptococcus salivarius* ^b^ ATCC 25975	200	100	25	25	0922
	*Streptococcus sobrinus* ^b^ ATCC 33478	200	200	25	25	1844
	*Streptococcus mitis* ^b^ ATCC 49456	100	100	50	50	3688
Cytotoxic activity	Vero cells ATCC CCL 81	456 ± 9	227 ± 16	>512	>512	-----

^a^ Gram-negative bacteria; ^b^ Gram-positive bacteria; * CHD = chlorhexidine dihydrochloride (positive control).

Seasonal variations exerted notable effects on the composition of the essential oils of *I. laurina* that reflected upon the antimicrobial activity. The essential oils, mainly of the rainy season, showed promising inhibition of the bacterial growth. Extracts or essential oils of plants with MIC values below 100 µg·mL^−1^ are considered promising as potential antimicrobial agents [34].

The essential oils from the bark and leaves of *I. laurina* of the rainy season inhibited the growth of anaerobic microorganisms with MIC values from 50 to 400 µg·mL^−1^, while for the essential oils obtained in the dry season, the values were from 100 to 200 µg·mL^−1^ (Table 5). Regarding the aerobic microorganisms, the essential oils from the bark and leaves of the dry season exhibited MIC values of 100 or 200 µg·mL^−1^ and during the rainy season, the essential oils exhibited the lowest inhibitory concentrations with MIC values of 25 or 50 µg·mL^−1^ (Table 5). These results indicate that the essential oils from bark and leaves extracted in the rainy season showed strong antimicrobial activity against all aerobic oral pathogens evaluated, emphasizing the results for inhibition of growth of *S. mutans*, principal etiological agent of dental caries, with MIC of 25 µg·mL^−1^. Therefore, the comparison of Table 2 and Table 4 can give an indication of the compounds responsible for the best results of inhibition observed for the essential oils of the rainy season; fatty acids appear as most likely. Studies have shown that fatty acids have antibacterial activity against many microorganisms [35]. It remains unclear exactly how fatty acids exert their antibacterial activities, but the prime target seems to be the bacterial cell membrane and the various essential processes that occur within and at the membrane [36]. Hydrophobic groups of fatty acids have shown a great influence on antimicrobial activity [37] because they allow interaction with hydrophobic proteins and lipids of the bacterial surface [38]. The antibacterial activity of fatty acids can be influenced by length of the carbon chain and the presence, number, position and orientation of double bonds [36]. It is possible that *I. laurina* synthesized fatty acids in the rainy season for your protection because, in this period, the humidity favors the proliferation of microorganisms. The terpenes can also be responsible for the inhibitory potential of oils of *I. laurina* since these compounds have shown activity against several oral microorganisms [39,40,41]. The significant antimicrobial activity of the essential oils of *I. laurina* may be related to compounds in higher concentration or synergistic interaction between major and minor compounds of the mixture.

Some compounds of the essential oil from the bark and leaf of *I. laurina* obtained in the rainy season have been reported in the literature for their recognized antimicrobial properties, such as (*Z*)-hex-3-en-1-ol (**15**) and linalool (**2**) [42], and lauric (**20**), linoleic (**23**) and palmitoleic acid [35], (*E*)-nerolidol [43], palmitic acid (**19**) [44] and phytol (**1**) [45]. In the essential oil from leaves, nonacosane (**16**) accounted for 21.95% and although no antimicrobial property has been reported in the literature, it is possible that this compound could have contributed to the low MIC values obtained.

Important results were also obtained for aerobic and anaerobic microorganisms in the dry season (Table 5). The essential oils of the leaves inhibited the growth of *P. gingivalis*, *P. nigrescens*, *S. sanguinis*, *S. salivarius* and *S. mitis* with MICs of 100 µg·mL^−1^. Values of MIC of 100 µg·mL^−1^ were also found for *P. gingivalis* and *S. mitis* when the essential oil of the bark was tested. The results obtained for essential oils of the dry season may be due to the presence of (*Z*)-hex-3-en-1-ol (14.23%) (**15**) in the leaves and linalool (5.17%) (**2**) and phytol (9.76%) (**1**) in the bark. Furthermore, in these essential oils, there was the presence of methyl salicylate (**14**), geraniol (**4**) and *γ*-eudesmol (**5**), which have antimicrobial properties when present in essential oils according to the literature [46,47,48].

The essential oils from leaves and bark of *I. laurina* exhibited relevant antibacterial activity against oral microorganisms showing MIC values lower than 100 μg·mL^−1^ and lower than others studies in the literature. The essential oil from the bark and leaves of *Cassia bakeriana* inhibited the growth of *S. mitis*, *S. sanguinis* and *S. mutans,* with MICs ranging from 62.5 μg·mL^−1^ to 125 μg·mL^−1^. The same values were found for anaerobic microorganisms, *B. fragilis* and *P. gingivalis* [22]. In another study, essential oils of *Campomanesia pubescens* inhibited the growth of *F. nucleatum*, *B. fragilis*, *S. sanguinis*, *S. mutans* and *S. mitis* with MICs in the range 62.5 μg·mL^−1^ to 2000 μg·mL^−1^ [21]. Essential oil of *Leptospermum scoparium*, *Melaleuca alternifolia*, *Eucalyptus radiata* and *Rosmarinus officinalis* inhibited the growth of *P. gingivalis*, *F. nucleatum*, *S. sobrinus* and *S. mutans* with MICs ranging from 300 μg·mL^−1^ to 10,000 μg·mL^−1^ [49]. The essential oil of *Artemisia iwayomogi* and standards of terpenes were tested against various oral pathogens showing MIC's range 800 to 12,800 μg·mL^−1^ [50]. In another study twenty essential oils of different plant were evaluated against *S. mutans*, the MIC’s ranged from 62.5 to 250 μg·mL^−1^ for most of the tested oils [51].

Cytotoxicity assays showed that all the tested oils had Cytotoxic Concentration (CC_50_) values above the minimum inhibitory concentrations. This is indicative that the oils have low toxicity at concentrations that inhibited microbial growth. A relationship between cytotoxicity and antimicrobial activity was established through the Selectivity Index (SI), which was calculated by the logarithm of the ratio of the CC_50_ and the MIC values for microorganisms (SI = log [CC_50_]/[MIC]). A positive value represents higher selectivity against microorganisms and low toxicity to Vero cells and a negative value indicates higher toxicity to Vero cells and low selectivity to the bacteria [52]. The SI for the essential oils from the bark and leaves in the rainy season at inhibitory concentration of 25 μg·mL^−1^, 50 μg·mL^−1^, 100 μg·mL^−1^ and 200 μg·mL^−1^ were above 1.31, 1.01, 0.71 and 0.41 respectively, once the cytotoxic concentrations of these samples are higher than 512 μg·mL^−1^. The SI for the essential oils extracted in the dry season at inhibitory concentration of 100 μg·mL^−1^ was 0.36 and 0.66 for leaves and bark respectively. For a concentration of 200 μg·mL^−1^, the SI was 0.36 and 0.05 for essential oils from bark and leaves respectively. All values of SI were positive, indicating that the essential oils from *I. laurina* showed higher antimicrobial activity than cytotoxicity.

## 3. Experimental

### 3.1. Plant Material and Essential Oil Extraction

Plant material was collected randomly from adult trees (approximately 6 m height) close to each other. The collection was done during the morning (8 a.m.), in two different seasons: in the months of June and July of 2012 (dry season) and in the months of November and December of 2012 (rainy season). The collection location has Aw climatic classification according to Köppen, dry winters and rainy summers. Leaves and stem bark of *I. laurina* were collected in the municipality of the Uberlândia City, Minas Gerais State, Brazil (18°59’13.96’’S; 48°12’42.16’’W). There was no separation between young and old leaves. The plant specimens were identified by a specialist, and a voucher specimen was deposited in the Herbarium of the Federal University of Uberlândia, under number 64050.

Fresh leaves and stem bark of *I. laurina* were cleaned, cut into small pieces and about 400 g of each part was individually put in round-bottomed flasks. Essential oil extraction was done by hydrodistillation using a Clevenger-type apparatus, over 4 h. The oil obtained was extracted with 5.0 mL of dichloromethane. The organic fraction was dried with anhydrous sodium sulphate, filtered and kept in a closed vial under refrigeration (−10 °C) for further analysis. The percentage yield was calculated relative to the dried mass of the initial sample.

### 3.2. Analysis and Identification of the Constituents

The oil was analysed by gas chromatography coupled to mass spectrometry (model GC17A/QP5010, Shimadzu, Uberlândia, Brazil), equipped with a SPC-5 capillary column (30 m × 0.25 mm × 0.25 μm film thickness). The carrier gas used was helium at a flow rate of 1 mL/min, detector and injector temperatures were 220 °C and 246 °C respectively, the injection volume was 1 µL and the split ratio was 1:20. The oven temperature was programmed from 60 °C to 246 °C at 3 °C/min. The electron impact energy was set at 70 eV and fragments from 40 to 650 *m*/*z* were collected.

The identification of the essential oil components was carried out by comparison of the mass spectrum obtained with those stored in the software libraries (Wiley7; Wiley229; Nist08; Nist08s; Nist27; Shim2205) and also by comparing the calculated arithmetic indices (AI) with the arithmetic indices reported in the literature [24,25,27]. Authentic standards were used when necessary. Our laboratory has a library of injected standards of natural products and some of them are present in the analyzed oils. They are marked with an asterisk (*) in Table 1 and Table 2. Arithmetic indices were calculated using equation AI (X) = 100 PzC + 100 [(t (X) − t (Pz))/(t (Pz + 1) − t (Pz))], which is based on retention times of linear alkane standards, which, by definition, have an AI equal to 100 × number of carbon atoms; X = compound at time t; PzC = number of carbon atoms of the alkane Pz, which runs just before X; Pz + 1 = alkane running after X [53]. Quantification was obtained after normalization of the peak areas in the total ion chromatogram (TIC). Results represent average values of three experiments.

### 3.3. Microbial Strains

The tested strains were obtained from American Type Culture Collection (ATCC, Rockville, MD, USA). The following microorganisms were used in the evaluation of the antibacterial activity of the essential oils: *Streptococcus mutans* (ATCC 25175), *Streptococcus sobrinus* (ATCC 33478), *Streptococcus sanguinis* (ATCC 10556), *Streptococcus salivarius* (ATCC 25975), *Streptococcus mitis* (ATCC 49456), *Actinomyces naeslundii* (ATCC 19039), *Porphyromonas gingivalis* (ATCC 33277), *Prevotella nigrescens* (ATCC 33563), *Bacteroides fragilis* (ATCC 25285) and *Fusobacterium nucleatum* (ATCC 25586).

### 3.4. Antimicrobial Activity

The minimum inhibitory concentration (MIC) values of the essential oils of different parts of *I. laurina* were determined in triplicate by the microdilution broth method in 96-well microplates (TPP^®^, EUA) [39]. The samples were dissolved in dimethyl sulfoxide (DMSO, Synth, São Paulo, Brazil; 8000 μg·mL^−1^), followed by dilution in tryptic soy broth (TSB, Difco, Detroit, MI, USA) for aerobic and Schaedler broth (Difco) supplemented with hemin (5.0 μg·mL^−1^) and vitamin K1 (10.0 μg·mL^−1^) for anaerobic, to achieve concentrations ranging from 400 to 12.5 μg·mL^−1^. The final DMSO concentration was 4% (*v*/*v*) and this solution was used as a negative control. The inoculum was adjusted for each organism to yield a cell concentration of 5 × 10^5^ colony forming units (CFU) per mL, according to the National Committee for Clinical Laboratory Standard (NCCLS) guidelines [54]. Chlorhexidine dihydrochloride (CHD, Sigma, Poole, Dorset, UK) was used as a positive control and the concentrations ranged from 0.0115 μg·mL^−1^ to 5.9 μg·mL^−1^. Controls of sterility of the TSB and Schaedler broths, control culture (inoculum), chlorhexidine dihydrochloride, essential oils and DMSO were performed. The microplates with the aerobic microorganisms were closed with a sterile plate sealer and incubated aerobically at 37 °C for 24 h. The anaerobic microorganisms were closed with a sterile plate sealer and incubated for 48–72 h in an anaerobic chamber (Don Whitley Scientific, Bradford, UK) in 5%–10% H_2_, 10% CO_2_, 80%–85% N_2_ atmosphere, at 37 °C. After that, resazurin (Sigma, 30 μL) in aqueous solution (0.01%) was added to indicate the viability of the microorganisms [39]. The MIC values were determined as the lowest concentration of essential oil capable of inhibiting the growth of the microorganisms.

### 3.5. Cytotoxic Activity

Samples of the essential oils were dissolved in methanol and diluted in culture medium DMEM supplemented to form a stock solution of 640 μg·mL^−1^. The cell viability test was performed with Vero cells (ATCC CCL 81; kidney epithelial cells of the African green monkey). For evaluation of cytotoxicity, the microplate dilution method was used. A solution containing 1 × 10^6^ cells in 10 μL supplemented with DMEM was prepared and 100 μL of this solution was pipetted into each well and then the plate was incubated for 6 h at 37 °C with humidified atmosphere and 5% CO_2_, allowing cell adhesion in the well. Once attached, the culture medium was removed and solutions of the samples were added at concentrations of 512, 256, 128, 64, 32, 16, 8 and 4 μg·mL^−1^, starting from the stock solution. The final volume in each well was 100 μL and the amount of cells present in each well was 1 × 10^4^. The final concentration of methanol in each well did not exceed 3%. For this analysis, the controls of cell growth, solvent, samples and the negative control (100% lysed cells) were performed. The microplates were incubated for 48 h at 37 °C with humidified atmosphere and 5% CO_2_. Next, 10 µL of revealing solution of resazurin (3 mM) diluted in PBS was added to each well [55] and the plate was incubated again for 24 h under the same conditions. Readings of absorbance at 594 nm were performed in a microplate spectrophotometer. The assays were carried out in triplicate and the results of the absorbance for each concentration tested were calculated according to the growth control. The EC_50_ (concentration at which 50% of the cells are viable) was calculated by a dose-response graph nonlinear regression [56].

### 3.6. Statistical Analysis

The essential oil yields are expressed as mean ± SD for analysis performed in triplicate. Statistical analysis of the data were performed by *t* test for yield comparisons of the essential oils and Analysis of Variance (ANOVA) followed by Tukey test for analysis of cytotoxic activity using SigmaPlot 11.0 software. Probability value *p* ≤ 0.05 was considered to denote a statistically significant difference.

## 4. Conclusions

Essential oils from the bark and leaves of *I. laurina*, extracted in dry and rainy seasons, presented a very small yield and large differences in quantitative and qualitative profile of volatile constituents. In general, all essential oils showed antimicrobial activity against aerobic and anaerobic microorganisms with bacteriostatic action, especially against *P. gingivalis*, *S. mutans*, *S. sanguinis*, *S. salivarius*, *S. sobrinus* and *S. mitis*. Essential oils extracted in the rainy season showed a better inhibition of the bacterial growth when compared to the oils of the dry season, particularly with respect to aerobic microorganisms. The large amount of fatty acids in the rainy season essential oils and the total absence of these compounds in the oils extracted in the dry season could be responsible for the better inhibitory effects observed. The essential oils of *I. laurina* indicated higher selectivity against oral pathogens and low toxicity to Vero cells. These results suggest that the essential oils of *I. laurina* are a source of biologically active compounds and may be a model for the development of antimicrobial agents.
